# Associations of aspirin and other anti-inflammatory medications with breast cancer risk by the status of COX-2 expression

**DOI:** 10.1186/s13058-022-01575-3

**Published:** 2022-12-09

**Authors:** Lusine Yaghjyan, A. Heather Eliassen, Graham Colditz, Bernard Rosner, Pepper Schedin, Akemi Wijayabahu, Rulla M. Tamimi

**Affiliations:** 1 Department of Epidemiology, College of Public Health and Health Professions and College of Medicine, University of Florida, 2004 Mowry Rd, Gainesville, FL 32610 USA; 2 Channing Division of Network Medicine, Department of Medicine, Brigham and Women’s Hospital, Harvard Medical School, Boston, MA USA; 3 Department of Surgery, Washington University in St. Louis School of Medicine, St. Louis, MO USA; 4 Institute for Public Health, Washington University in St. Louis, St. Louis, MO USA; 5 Oregon Health and Science University, Portland, OR USA; 6 Department of Population Health Sciences, Weill Cornell Medicine, New York, NY USA

**Keywords:** Breast cancer risk, Aspirin, COX-2, NSAIDs

## Abstract

**Background:**

We investigated the associations of aspirin and other nonsteroidal anti-inflammatory drugs (NSAIDs) with breast cancer risk by the status of COX-2 protein expression.

**Methods:**

This study included 421 cases and 3,166 controls from a nested case–control study within the Nurses’ Health Study (NHS) and Nurses’ Health Study II (NHSII) cohorts. Information on medication use was first collected in 1980 (NHS) and 1989 (NHSII) and was updated biennially. Medication use was defined as none, past or current; average cumulative dose and frequency were calculated for all past or current users using data collected from all biannual questionnaires preceding the reference date. Immunochemistry for COX-2 expression was performed using commercial antibody (Cayman Chemical and Thermo Fisher Scientific). We used polychotomous logistic regression to quantify associations of aspirin and NSAIDs with the risk of COX2+ and COX2− breast cancer tumors, while adjusting for known breast cancer risk factors. All tests of statistical significance were two-sided.

**Results:**

In multivariate analysis, we found no differences in associations of the aspirin exposures and NSAIDs with breast cancer risk by COX2 expression status. In stratified analyses by COX2 status, significant associations of these medications with breast cancer risk were observed for dosage of aspirin among current users in COX2- tumors (OR for > 5 tablets per week vs. none 1.71, 95% CI 1.01–2.88, p-trend 0.04). Regular aspirin use was marginally associated with the risk of COX2- tumors (p-trend = 0.06).

**Conclusions:**

Our findings suggested no differences in associations of aspirin and other NSAIDs with COX2+ and COX2− tumors.

**Supplementary Information:**

The online version contains supplementary material available at 10.1186/s13058-022-01575-3.

## Background

Chronic inflammation is a known risk factor for several cancers [1] and is a complex biological process which helps to promote cell division and repair at the injured tissue site [2]. In previous studies, increased levels of circulating inflammatory markers have been associated with an increased risk of various cancers [3]. At the same time, chronic inflammation can stimulate angiogenesis, prevent apoptosis, and promote proliferation and metastatic spread [2]. Several markers of chronic inflammation have been previously linked to an increased breast cancer risk, though the overall evidence on the role of inflammation in etiology of breast cancer remains largely inconsistent [2, 3].

Epidemiologic studies on the association between nonsteroidal anti-inflammatory drugs (NSAIDs) and, specifically, aspirin use and breast cancer demonstrated inconsistent findings. While some studies found no association [4–8], other studies demonstrated an inverse association between aspirin and breast cancer [9–15], and between aspirin and mammographic breast density, a strong breast cancer risk factor [16]. A meta-analysis of 38 studies found that use of nonsteroidal anti-inflammatory drugs (NSAIDs) was associated with a 12% reduced risk of breast cancer and in the aspirin-specific analysis, a 13% reduction in breast cancer risk [17]. A potential biological mechanism through which aspirin may reduce breast density and breast cancer risk is via the inhibition of cyclooxygenase-2 (COX-2) enzyme activity [7, 13, 14]. COX-2 enzyme mediates the synthesis of prostaglandin E2 (PGE-2) [7], which modulates apoptosis and cell proliferation [13] and may influence endogenous estrogen levels through the stimulation of aromatase [7]. Consequently, through the suppression of COX-2, aspirin may lower PGE-2 production, thereby reducing its pro-tumorigenic activity in mammary cells, and inhibiting tumor growth [7, 13].

It is unknown whether the associations of anti-inflammatory medications, including aspirin, with breast cancer may differ across COX-2 defined breast tumor subtypes. In this study, we aimed to compare the associations of aspirin and other NSAIDs with COX-2 positive and COX-2 negative tumors using Nurses’ Health Study (NHS) and the Nurses’ Health Study II (NHS II) cohorts.

## Methods

### Study population and design

Women included in this study were selected from participants of the NHS and NHSII cohorts. These prospective cohorts followed registered nurses in the USA who were 30–55 years (NHS) or 25–42 years old (NHSII) at enrollment. After administration of the initial questionnaire, the information on breast health risk factors and any cancer diagnoses was updated biennially. More detailed description of the cohort has been published elsewhere [18].

We used a nested case–control study design to examine the association between aspirin and other NSAIDs with breast tumor subtypes defined based on the COX-2 expression status. Details of this nested case–control study have been previously described [19]. Briefly, using incidence density sampling, women who did not have any type of cancer at the time of the cases’ breast cancer diagnosis (controls) were matched with women diagnosed with in situ or invasive breast cancer (cases) during the follow-up period from June 1, 1989, through June 30, 2004, for NHS and from June 1, 1996, to June 30, 2007, for NHSII [18]. Breast cancer cases were confirmed through medical record review by trained personnel. Because the original study was designed to evaluate associations between circulating biomarkers and breast cancer risk, the cases were matched with controls on age, menopausal status, postmenopausal hormone use (current vs. not current) at blood collection, and day and time of blood collection. We restricted our analysis to 421 cases and 3,166 controls with available data on COX-2 status and important covariates, including mammographic breast density, a well-established and strong breast cancer risk factor.

The study protocol was approved by the institutional review boards of the Brigham and Women’s Hospital and Harvard T.H. Chan School of Public Health, and those of participating registries as required. Consent was obtained or implied by return of questionnaires.

### Assessment of aspirin intake and NSAIDs

The methods of assessing exposure to aspirin and other NSAIDs have been described in detail elsewhere [20]. Briefly, information on aspirin use in NHS was first obtained in 1980 and biennially thereafter except in 1986. In 1980, participants were asked whether they currently took aspirin in most weeks and, if yes, what was the weekly amount and years of aspirin use. Information on aspirin dose and frequency of use was also collected beginning in 1982 and 1984, respectively. In NHSII, on the baseline questionnaire in 1989, participants were asked if they regularly (≥ 2 times per week) used aspirin, or other anti-inflammatory drugs in three separate questions and this was updated biennially from 1993. Beginning in 1993 (for aspirin) or 1995 (for other anti-inflammatory drugs), women were asked to report frequency of use (categorized as either days per week or days per month). Beginning in 1999, participants were additionally asked about quantity used (tablets per week) in each category.

Women were classified as current users at each questionnaire in which current use was reported and were considered current users for the subsequent two-year follow-up period (or the 4-year follow-up period from 1989 to 1993). For participants who missed a questionnaire, drug use information was carried forward from the previous cycle. The women who ceased reporting use were classified as past users, but they were eligible to become current users in subsequent follow-up years. Women were classified as nonusers if they did not report analgesic use at baseline or on any of their follow-up questionnaires. Duration of use of each drug was calculated from baseline (1980 for aspirin, 1990 for other NSAIDs for NHS, and 1989 for NHSII) to the reference date (date of diagnosis for cases and their matched controls) [20]. To better represent long-term use, we calculated the cumulative average dose (standard 325-mg tablet) and frequency (days per week) for each woman who was classified as a past or current user as the average of current use and all previous follow-up cycles. Status, quantity, and frequency of use were carried forward one cycle to replace missing data, and cumulative average quantity, cumulative average frequency, and duration of use were calculated from these variables with the carried-forward data.

### Tumor tissue analyses

We requested formalin-fixed paraffin-embedded tissue samples from hospitals throughout the US where women underwent primary breast tumor resection. Tumor microarrays (TMAs) were constructed at the Dana Farber Harvard Cancer Center Tissue Microarray Core Facility, Boston, MA. As described previously [21], TMAs were assembled by taking three 0.6-mm-diameter cores from each breast cancer sample and inserting cores into a recipient TMA block.

Immunohistochemistry for COX-2 was conducted on 5 μm paraffin sections of TMA blocks. Cores with fewer than 100 cells were excluded from all analyses. For NHS, the staining was first performed with monoclonal antibodies from Thermo Fisher Scientific (SP21 clone, Waltham, Massachusetts, USA). These stained NHS TMAs were subsequently stained with a second monoclonal antibody from Cayman Chemical (CX229 clone, Ann Arbor, Michigan, USA) at the Pepper Schedin’s laboratory (Oregon Health and Science University, Portland, Oregon, USA), as previously described [22].

Expression of COX-2 for NHSII TMAs was assessed in Dr. Pepper Schedin’s laboratory (Oregon Health and Science University, Portland, OR) [22]. Based on previous reports of differing COX-2 staining patterns for the monoclonal antibodies produced by Cayman Chemical (CX229 clone, Ann Arbor, Michigan, USA) and Thermo Fisher Scientific (SP21 clone, Waltham, Massachusetts, USA) [23], TMA slides were dual-stained at a single time point using both antibodies and labeled with distinct chromogens so that the two COX-2 signals could be distinguished. COX-2 staining results from both NHS and NHSII TMAs were analyzed using the Aperio co-localization image analysis algorithm and expressed as percentages of positively stained area for each antibody. In primary analyses, we examined the mean % area across all three cores that stained positive for at least one of the two antibodies. In supplemental analyses, we examined the mean % area for each antibody separately. Tumor expression status was defined as either negative or positive using the median % positivity as a cutoff.

### Covariate information

Information on breast cancer risk factors was obtained from the biennial questionnaires closest to the date of the mammogram. Women were considered to be postmenopausal if they reported: (1) no menstrual periods within the 12 months before blood collection with natural menopause, (2) bilateral oophorectomy, or (3) hysterectomy with one or both ovaries retained and were 54 years or older for ever-smokers or 56 years or older for never-smokers [24, 25].

To quantify mammographic density, the craniocaudal views of both breasts for all screening mammograms in the NHS and for the first two batches of mammograms in the NHSII were digitized at 261 μm per pixel with a Lumisys 85 laser film scanner (Lumisys, Sunnyvale, California). The third batch of NHSII mammograms was digitized using a VIDAR CAD PRO Advantage scanner (VIDAR Systems Corporation; Herndon, VA) and comparable resolution of 150 dots per inch and 12 bit depth. The Cumulus software (University of Toronto, Toronto, Canada) was used for computer-assisted determination of the absolute dense area, non-dense area, and percent mammographic density on all mammograms [26, 27]. Percent breast density was measured as percentage of the total area occupied by epithelial/stromal tissue (absolute dense area) divided by the total breast area. Because breast densities of the right and left breast for an individual woman are strongly correlated [26], the average density of both breasts was used in this analysis.

### Statistical analysis

We used unconditional logistic regression to analyze the association between anti-inflammatory drug use and breast cancer risk, while adjusting for the following potential confounders in the fully adjusted logistic regression models: age at diagnosis (continuous, years), body mass index (BMI, continuous, kg/m^2^), percent breast density (< 10%, 10 to  < 25%, 25 to  < 50%, ≥ 50%), age at menarche (< 12, 12, 13, > 13 years), parity and age at first birth (nulliparous, parous/ < 25 years, and parous/ ≥ 25 years), PMH use (never, ever, unknown), family history of breast cancer (yes, no), alcohol consumption (0, < 5, 5 to < 15, ≥ 15 g/day), and study cohort (NHS, NHSII).

Differences in the association of breast density with COX2-defined tumor subtypes were investigated using polychotomous logistic regression [27]. In this analysis, the outcome has three levels which include controls and two breast cancer subtypes defined based on the COX2 status (positive and negative). We used a likelihood ratio test to compare a model with separate anti-inflammatory drug use slopes in each case group with a model with a common slope. This method has been described in detail elsewhere [19]. In this analysis, the drug intake variables were modeled using respective medians within each of the categories. For all analyses, the level of statistical significance was assessed at *α* equal to 0.05. All tests were two-sided. All analyses except the test of heterogeneity were performed using SAS software (version 9.2, SAS Institute, Cary, NC, USA). The test of heterogeneity from polychotomous logistic regression models was done using STATA version 11.0 (Stata Corp, College Station, TX, USA).

## Results

Our study included 421 cases and 3,166 controls. Age-adjusted characteristics of controls in the study by menopausal status and aspirin intake have been described previously [28]. Based on primary staining method (using staining results from both antibodies), our sample included 227 COX2+ and 194 COX2− tumors. Based on the secondary staining approaches, we had 214 COX2+ and 207 COX2− tumors for Cayman antibody staining method and 223COX+ and 198 COX2− tumors with Thermo Scientific antibody staining.

In the primary analysis using staining from both antibodies for COX2 (Table 1), we found no differences in associations of the aspirin exposure variables as well as NSAIDs with breast cancer risk by COX2 protein expression status. Dosage of aspirin among current users was positively associated with the risk of COX2− tumors (OR for > 5 tablets per week vs. none 1.71, 95% CI 1.01–2.88, p-trend 0.04) and marginally associated with the risk of COX2+ tumors (p-trend = 0.07, p-heterogeneity = 0.90). Regular aspirin use was marginally associated with the risk of COX2− tumors (p-trend = 0.06).

**Table 1:** Associations of aspirin with breast cancer risk, by COX2 status defined using median, combined staining with Cayman and Thermo Scientific antibodies

Intake category	COX2 negative^a^		COX2 positive^a^		p-heterogeneity
	*N*	Full models, OR and 95% CI	*N*	Full models, OR and 95% CI	
**Aspirin use**					
Regular use (≥ 2 times/week)					
Nonusers	50/1102	Ref	102/1102	Ref	
Past users	56/946	0.98 (0.63, 1.52)	60/946	1.18 (0.82, 1.70)	
Current users					
< 5 years	13/228	1.21 (0.64, 2.30)	15/228	0.76 (0.43, 1.35)	
≥ 5 years	75/890	1.39 (0.89, 2.17)	50/890	1.32 (0.88, 2.00)	
p-trend		0.06		0.42	0.41
Frequency of use					
Nonusers	50/1038	Ref	102/1038	Ref	
Past users	69/1057	1.00 (0.66, 1.52)	62/1057	1.06 (0.74, 1.52)	
Current users					
1 day/week	34/277	1.72 (1.04, 2.85)	8/277	0.55 (0.26, 1.17)	
2–3 days/week	17/206	1.20 (0.65, 2.20)	19/206	1.57 (0.91, 2.71)	
4–5 days/week	9/159	0.95 (0.44, 2.04)	11/159	1.47 (0.74, 2.90)	
6+ days/week	43/598	1.26 (0.78, 2.03)	31/598	1.05 (0.66, 1.67)	
p-trend		0.92		0.73	0.74
Dosage (number of tablets per week)					
Nonusers	52/798	Ref	76/798	Ref	
Past users					
< 2	29/437	1.01 (0.59, 1.73)	20/437	0.94 (0.51, 1.74)	
2–5	9/164	0.73 (0.32, 1.66)	6/164	1.12 (0.48, 2.62)	
> 5	4/84	0.35 (0.08, 1.50)	2/84	1.29 (0.43, 3.86)	
p-trend		0.11		0.77	0.15
Current users					
< 2	25/350	1.30 (0.77, 2.19)	17/350	0.54 (0.29, 1.03)	
2–5	22/330	1.08 (0.60, 1.94)	16/330	1.07 (0.58, 1.96)	
> 5	33/343	1.71 (1.01, 2.88)	30/343	1.56 (0.95, 2.57)	
p-trend		0.04		0.07	0.90
Duration (years of use by status)					
Nonusers	50/1102	Ref	102/1102	Ref	
Past users					
< 2	0/23	< 0.00 (< 0.00, > 999.99)	2/23	1.54 (0.34, 6.87)	
2–5	23/355	1.17 (0.69, 1.99)	29/355	1.07 (0.69, 1.66)	
> 5	33/568	0.87 (0.52, 1.46)	29/568	1.37 (0.84, 2.24)	
p-trend		0.67		0.57	0.47
Current users					
< 2	7/105	1.56 (0.68, 3.58)	5/105	0.54 (0.21, 1.37)	
2–5	8/146	1.11 (0.51, 2.42)	12/146	0.93 (0.49, 1.77)	
> 5	73/867	1.33 (0.85, 2.08)	48/867	1.42 (0.93, 2.16)	
p-trend		0.28		0.18	0.86
**NSAIDs**					
Regular use (≥ 2 times/week)					
Nonusers	85/1177	Ref	61/1177	Ref	
Past users	53/856	0.94 (0.65, 1.35)	71/856	1.20 (0.83, 1.73)	
Current users					
< 5 years	52/605	1.09 (0.75, 1.58)	30/605	0.79 (0.50, 1.25)	
≥ 5 years	34/788	0.72 (0.47, 1.11)	70/788	1.10 (0.75, 1.61)	
p-trend		0.31		0.94	0.48
Frequency of use					
Nonusers	85/1092	Ref	61/1092	Ref	
Past users	53/851	0.89 (0.62, 1.29)	71/851	1.03 (0.71, 1.49)	
Current users					
1 day/week	28/427	0.80 (0.50, 1.27)	31/427	0.81 (0.51, 1.29)	
2–3 days/week	23/404	0.82 (0.50, 1.35)	43/404	1.09 (0.71, 1.68)	
4–5 days/week	10/116	1.28 (0.63, 2.59)	11/116	1.00 (0.50, 2.00)	
6+ days/week	19/258	1.02 (0.59, 1.74)	13/258	0.80 (0.42, 1.51)	
p-trend		0.80		0.93	0.81

Adjusted for age (continuous), BMI (continuous), percent breast density ((< 10%, 10 to  < 25%, 25 to  < 50%, ≥ 50%), age at menarche (< 12, 12, 13, > 13), a family history of breast cancer (Yes/No), a history of benign breast disease (Yes/No), NHS cohort (NHSI, NHSII), alcohol use (none, > 0 to  < 5, ≥ 5 g/day), menopausal status/postmenopausal hormone use (premenopausal, postmenopausal/no hormones, postmenopausal/past hormones, postmenopausal/current hormones, postmenopausal/past/unknown current), and parity and age at first child’s birth (nulliparous, parous with age at first birth < 25, parous with age at first birth ≥ 25)

^a^Positive versus negative COX-2 status was defined using the median % of positive staining for at least one of the two antibodies (Cayman Chemical or Thermo Fisher Scientific)

In the secondary analysis using either Cayman staining antibody or Thermo Scientific antibody staining-defined COX-2 status (Additional file 1: Tables 1 and 2, respectively), we observed no differences in associations of the aspirin exposure variables and NSAIDs across Cayman staining antibody-defined or Thermo Scientific antibody-defined COX2+ and COX− tumors. With Thermo Scientific staining antibody, regular aspirin use and dosage among current users were associated with an increased risk of COX2+ tumors (p-trend = 0.04 for both) and duration among current users was marginally associated with an increased risk of COX2+ tumors (p-trend = 0.05). None of the anti-inflammatory medications were associated with the risk of COX2− tumors defined with either of the two secondary staining approaches.

## Discussion

In this study of associations of anti-inflammatory drug use and breast cancer by the status of COX-2 protein expression, we found no differences in the association patterns of aspirin or other NSAIDs across COX2-defined tumor subtypes.

The findings of the studies on associations of aspirin with breast cancer risk have been inconsistent with some suggesting an inverse associations of aspirin use with breast cancer and breast cancer-specific mortality after primary breast cancer diagnosis [9–15] and others finding no associations [4, 7, 8, 20]. The existing evidence on these associations has recently been summarized by a meta-analysis of 38 studies [17]. Use of any NSAIDs was associated with a 12% reduced risk of breast cancer (relative risk [RR] = 0.88, 95% confidence interval [CI] 0.84–0.93), with similar associations seen for aspirin use (RR = 0.87, 95% CI 0.82–0.92) and stronger associations for ibuprofen (RR = 0.79, 95% CI 0.64–0.97) [17]. Two studies of associations between anti-inflammatory medication and breast cancer in NHS and NHSII found no associations in both pre- and postmenopausal women [20, 29].

Several biological mechanisms were suggested as a possible explanation for potential effects of aspirin and other anti-inflammatory medications on breast cancer risk, including inhibition of COX-2 enzyme activity [7, 13, 14] that mediates the synthesis of prostaglandin E2 (PGE-2) [7]. PGE-2 is known to modulate apoptosis and cell proliferation [13] and to stimulate aromatase [7] which could in turn influence endogenous estrogens levels. Potential importance of this mechanism is further supported by studies demonstrating overexpression of COX-2 in women with mammary tumors when compared to women with normal breast tissue; however, the possibility of reverse causality cannot be rules out in these studies [7, 13]. Thus, aspirin can potentially lower PGE-2 production via suppression of COX-2 and prevent its pro-tumorigenic influences on mammary cells [7, 13]. In our study, even though the majority of heterogeneity tests showed statistical significance, there was no clear pattern in associations of any of the exposure variables with the risk of COX2+ versus COX2− subtypes defined using staining from both antibodies simultaneously. Dosage of aspirin among current users was positively associated with the risk of COX2− tumors and marginally associated with the risk of COX2+ tumors, while regular aspirin use was marginally associated with the risk of COX2− tumors. In the secondary analysis using either Cayman or Thermo Scientific staining antibodies-defined COX2 status, no differences in association patterns were observed across Cayman staining antibody-defined COX2+ and COX2− tumors. With Thermo Scientific staining antibody, regular aspirin use and dosage among current users were associated with an increased risk of COX2+ tumors and duration among current users was marginally associated with an increased risk of COX2+ tumors. Thus, our findings do not support the hypothesis that associations of anti-inflammatory medications with breast cancer risk are distinctly different in COX2+ versus COX2− tumors.

We examined, for the first time, associations of anti-inflammatory medications with the risk of COX2-defined breast tumor subtypes. Our study used data from the NHS and NHSII cohorts with more than 25 years of follow-up, ascertainment of disease status, and comprehensive information on breast cancer risk factors and breast density. Our study has a few limitations. Despite the prospective nature of the cohort, potential errors in recall of aspirin and other NSAIDs use are possible. However, given our population of registered nurses with a familiarity of health-related exposures and use of drugs as well as prospective data collection, the medication use data are likely to be accurate.

## Conclusions

We investigated the associations of aspirin and other NSAIDs use with breast cancer risk by the status of COX-2 protein expression. Our findings suggested no differences in associations of these medications with COX2+ and COX2− tumors.

## Supplementary Information

**Additional file 1. Supplementary table 1**. Associations of aspirin with breast cancer risk, by COX2 status defined using median Cayman staining antibody.** Supplementary table 2**. Associations of aspirin with breast cancer risk, by COX2 status defined using median, Thermo Scientific staining antibody.

## Data Availability

The data that support the findings of this study are available from the Nurses’ Health Studies; however, they are not publicly available. Investigators interested in using the data can request access, and feasibility will be discussed at an investigators’ meeting. Limits are not placed on scientific questions or methods, and there is no requirement for co-authorship. Additional data sharing information and policy details can be accessed at http://www.nurseshealthstudy.org/researchers.

## Abbreviations

BMI: Body mass index
CI: Confidence interval
NHS: Nurses’ Health Study
NSAIDs: Nonsteroidal anti-inflammatory drugs
OR: Odds ratio
TMA: Tissue microarray
