# Supplementary material for: Associations of aspirin and other anti-inflammatory medications with breast cancer risk by the status of COX-2 expression
Source: Breast Cancer Res. 2022 Dec 9;24:89. doi: 10.1186/s13058-022-01575-3 (PMC9733081; doi:10.1186/s13058-022-01575-3)
Supplement: Supplementary file 1 — Additional file 1. Supplementary table 1. Associations of aspirin with breast cancer risk, by COX2 status defined using median Cayman staining antibody. Supplementary table 2. Associations of aspirin with breast cancer risk, by COX2 status defined using median, Thermo Scientific staining antibody. [file 13058_2022_1575_MOESM1_ESM.doc]

**Supplementary table 1.** Associations of aspirin with breast cancer risk, by COX2 status defined using median Cayman staining antibody **^a^**

| **Intake category** | **COX2 negative** | | **COX2 positive** | | **p-heterogeneity** |
| --- | --- | --- | --- | --- | --- |
|  | **N**  **(Cases/**  **Controls** | **Full Models,**  **OR and 95% CI** | **N**  **(Cases/**  **Controls)** | **Full Models,**  **OR and 95% CI** |  |
| **ASPIRIN USE** |  |  |  |  |  |
| Regular use (≥2 times/week)  Non-users  Past users  Current users  <5 yrs  ≥5 yrs  p-trend | 61/1102  63/946  12/228  71/890 | Ref  1.12 (0.75, 1.68)  0.98 (0.51, 1.86)  1.36 (0.89, 2.09)  0.18 | 91/1102  53/946  16/228  54/890 | Ref  1.07 (0.73, 1.57)  0.90 (0.51, 1.57)  1.41 (0.93, 2.14)  0.17 | 0.98 |
| Frequency of use  Non-users  Past users  Current users  1 day/wk  2-3 days/wk  4-5 days/wk  6+ days/wk  p-trend | 61/1038  73/1057  23/277  21/206  9/159  43/598 | Ref  1.07 (0.72, 1.58)  1.18 (0.69, 2.02)  1.50 (0.86, 2.61)  0.94 (0.44, 2.01)  1.23 (0.78, 1.94)  0.70 | 91/1038  58/1057  19/277  15/206  11/159  31/598 | Ref  0.99 (0.68, 1.44)  1.26 (0.73, 2.18)  1.22 (0.67, 2.21)  1.48 (0.75, 2.93)  1.07 (0.67, 1.71)  0.88 | 0.69 |
| Dosage (number of tablets per week)  Non-users  Past users  <2  2-5  >5  p-trend  Current users  <2  2-5  >5  p-trend | 52/798  29/437  9/164  4/84  25/350  22/330  33/343 | Ref  0.87 (0.51, 1.49)  0.78 (0.36, 1.69)  0.67 (0.23, 1.97)  0.60  1.00 (0.59, 1.70)  0.98 (0.55, 1.74)  1.45 (0.87, 2.41)  0.10 | 76/798  20/437  6/164  2/84  17/350  16/330  30/343 | Ref  1.06 (0.59, 1.91)  0.91 (0.37, 2.25)  0.58 (0.14, 2.53)  0.30  0.80 (0.45, 1.42)  1.07 (0.58, 1.98)  1.77 (1.08, 2.92)  0.03 | 0.60  0.69 |
| Duration (years of use by status)  Non-users  Past users  <2  2-5  >5  p-trend  Current users  <2  2-5  >5  p-trend | 61/1102  0/23  27/355  36/568  4/105  9/146  70/867 | Ref  <0.00 (<0.00, >999.99)  1.28 (0.79, 2.07)  1.05 (0.64, 1.73)  0.67  0.75 (0.27, 2.13)  1.09 (0.52, 2.27)  1.37 (0.88, 2.11)  0.24 | 91/1102  2/23  25/355  26/568  8/105  11/146  51/867 | Ref  1.64 (0.37, 7.31)  1.01 (0.63, 1.62)  1.11 (0.66, 1.86)  0.79  0.94 (0.44, 2.02)  0.96 (0.49, 1.86)  1.42 (0.92, 2.19)  0.20 | 0.61  0.93 |
| **NSAIDs** |  |  |  |  |  |
| Regular use (≥2 times/week)  Non-users  Past users  Current users  <5 yrs  ≥5 yrs  p-trend | 82/1177  51/856  46/605  51/788 | Ref  0.87 (0.60, 1.27)  0.99 (0.67, 1.46)  0.98 (0.66, 1.44)  0.99 | 64/1177  73/856  36/605  53/788 | Ref  1.26 (0.88, 1.81)  0.92 (0.60, 1.42)  0.90 (0.60, 1.35)  0.34 | 0.48 |
| Frequency of use  Non-users  Past users  Current users  1 day/wk  2-3 days/wk  4-5 days/wk  6+ days/wk  p-trend | 82/1092  51/851  29/427  32/404  9/116  21/258 | Ref  0.80 (0.55, 1.17)  0.80 (0.50, 1.26)  1.00 (0.64, 1.57)  1.00 (0.48, 2.08)  1.11 (0.66, 1.87)  0.63 | 64/1092  73/851  30/427  34/404  12/116  11/258 | Ref  1.12 (0.78, 1.61)  0.82 (0.51, 1.30)  0.96 (0.61, 1.52)  1.21 (0.62, 2.37)  0.68 (0.35, 1.34)  0.71 | 0.53 |

**^a^** Adjusted for age (continuous), BMI (continuous), percent breast density ((<10%, 10-<25%, 25-<50%, ≥50%), age at menarche (<12, 12, 13, >13), a family history of breast cancer (Yes/No), a history of benign breast disease (Yes/No), NHS cohort (NHSI, NHSII), alcohol use (none, >0-<5, ≥5 g/day), menopausal status/postmenopausal hormone use (premenopausal, postmenopausal/no hormones, postmenopausal/past hormones, postmenopausal/current hormones, postmenopausal/past/ unknown current) and parity and age at first child’s birth (nulliparous, parous with age at first birth <25, parous with age at first birth ≥25)

**Supplementary table 2. Associations of aspirin with breast cancer risk, by COX2 status defined using median, Thermo Scientific staining antibody ^a^**

| **Intake category** | **COX2 negative** | | **COX2 positive** | | **p-heterogeneity** |
| --- | --- | --- | --- | --- | --- |
|  | **N**  **(cases/**  **controls)** | **Full Models,**  **OR and 95% CI** | **N**  **(cases/**  **controls)** | **Full Models,**  **OR and 95% CI** |  |
| **ASPIRIN USE** |  |  |  |  |  |
| Regular use (≥2 times/week)  Non-users  Past users  Current users  <5 yrs  ≥5 yrs  p-trend | 60/1102  63/946  14/228  61/890 | Ref  1.11 (0.74, 1.67)  1.12 (0.61, 2.05)  1.17 (0.75, 1.81)  0.52 | 92/1102  53/946  14/228  64/890 | Ref  1.05 (0.72, 1.53)  0.79 (0.44, 1.42)  1.63 (1.09, 2.43)  0.04 | 0.33 |
| Frequency of use  Non-users  Past users  Current users  1 day/wk  2-3 days/wk  4-5 days/wk  6+ days/wk  p-trend | 60/1038  75/1057  30/277  17/206  7/159  36/598 | Ref  1.10 (0.74, 1.63)  1.54 (0.93, 2.55)  1.18 (0.65, 2.14)  0.77 (0.33, 1.77)  1.05 (0.65, 1.69)  0.49 | 92/1038  56/1057  12/277  19/206  13/159  38/598 | Ref  0.94 (0.64, 1.36)  0.79 (0.42, 1.50)  1.58 (0.91, 2.72)  1.66 (0.87, 3.15)  1.25 (0.81, 1.95)  0.41 | 0.26 |
| Dosage (number of tablets per week)  Non-users  Past users  <2  2-5  >5  p-trend  Current users  <2  2-5  >5  p-trend | 50/798  36/437  7/164  2/84  25/350  17/330  31/343 | Ref  1.22 (0.72, 2.05)  0.68 (0.29, 1.61)  0.39 (0.09, 1.67)  0.10  1.09 (0.64, 1.87)  0.85 (0.46, 1.59)  1.50 (0.89, 2.53)  0.09 | 78/798  13/437  8/164  4/84  17/350  21/330  32/343 | Ref  0.59 (0.30, 1.14)  1.03 (0.46, 2.30)  0.98 (0.33, 2.91)  0.78  0.73 (0.41, 1.29)  1.25 (0.71, 2.20)  1.69 (1.04, 2.75)  0.04 | 0.14  0.81 |
| Duration (years of use by status)  Non-users  Past users  <2  2-5  >5  p-trend  Current users  <2  2-5  >5  p-trend | 60/1102  0/23  27/355  36/568  7/105  9/146  59/867 | Ref  <0.00 (<0.00, >999.99)  1.28 (0.79, 2.08)  1.01 (0.61, 1.65)  0.88  1.27 (0.56, 2.88)  1.09 (0.52, 2.28)  1.12 (0.71, 1.75)  0.64 | 92/1102  2/23  25/355  26/568  5/105  11/146  62/867 | Ref  1.64 (0.37, 7.28)  1.00 (0.63, 1.60)  1.11 (0.66, 1.84)  0.97  0.60 (0.24, 1.53)  0.93 (0.48, 1.81)  1.72 (1.14, 2.60)  0.05 | 0.89  0.31 |
| **NSAIDs** |  |  |  |  |  |
| Regular use (≥2 times/week)  Non-users  Past users  Current users  <5 yrs  ≥5 yrs  p-trend | 79/1177  56/856  51/605  40/788 | Ref  0.99 (0.69, 1.43)  1.11 (0.76, 1.62)  0.79 (0.52, 1.20)  0.46 | 67/1177  68/856  31/605  64/788 | Ref  1.12 (0.78, 1.61)  0.79 (0.50, 1.23)  1.03 (0.71, 1.51)  0.76 | 0.74 |
| Frequency of use  Non-users  Past users  Current users  1 day/wk  2-3 days/wk  4-5 days/wk  6+ days/wk  p-trend | 79/1092  56/851  30/427  26/404  10/116  20/258 | Ref  0.92 (0.64, 1.33)  0.83 (0.53, 1.32)  0.86 (0.53, 1.39)  1.18 (0.58, 2.39)  1.08 (0.63, 1.83)  0.76 | 67/1092  68/851  29/427  40/404  11/116  12/258 | Ref  0.98 (0.68, 1.42)  0.76 (0.48, 1.22)  1.06 (0.68, 1.63)  1.03 (0.52, 2.05)  0.72 (0.38, 1.38)  0.86 | 0.72 |

**^a^** Adjusted for age (continuous), BMI (continuous), percent breast density (<10%, 10-<25%, 25-<50%, ≥50%), age at menarche (<12, 12, 13, >13), a family history of breast cancer (Yes/No), a history of benign breast disease (Yes/No), NHS cohort (NHSI, NHSII), alcohol use (none, >0-<5, ≥5 g/day), menopausal status/postmenopausal hormone use (premenopausal, postmenopausal/no hormones, postmenopausal/past hormones, postmenopausal/current hormones, postmenopausal/past/ unknown current) and parity and age at first child’s birth (nulliparous, parous with age at first birth <25, parous with age at first birth ≥25)
